# Brain serotonergic and dopaminergic modulators, perceptual responses and endurance exercise performance following caffeine co-ingested with a high fat meal in trained humans

**DOI:** 10.1186/1550-2783-7-22

**Published:** 2010-05-27

**Authors:** Marios P Hadjicharalambous, Liam P Kilduff, Yannis P Pitsiladis

**Affiliations:** 1 Department of Life and Health Sciences, University of Nicosia, Nicosia, Cyprus; 2 Integrative and Systems Biology, Faculty of Biomedical and Life Sciences (FBLS), University of Glasgow, Glasgow, UK; 3 Sport and Exercise Science Research Centre, Vivian Tower, Swansea University, UK

## Abstract

**Background:**

The present study examined putative modulators and indices of brain serotonergic and dopaminergic function, perceptual responses, and endurance exercise performance following caffeine co-ingested with a high fat meal.

**Methods:**

Trained humans (n = 10) performed three constant-load cycling tests at 73% of maximal oxygen uptake (VO_2_max) until exhaustion at 10°C remove space throughout. Prior to the first test, subjects consumed a 90% carbohydrate meal (Control trial) and for the remaining two tests, a 90% fat meal with (FC trial) and without (F trial) caffeine.

**Results:**

Time to exhaustion was not different between the F and FC trials (*P *> 0.05); [Control trial: 116(88-145) min; F trial: 122(96-144) min; FC trial: 127(107-176) min]. However, leg muscular discomfort during exercise was significantly lower on the FC relative to F trial (*P *< 0.01). There were no significant differences between F and FC trials in key modulators and indices of brain serotonergic (5-HT) and dopaminergic (DA) function [(i.e. plasma free and total tryptophan (Trp), tyrosine (Tyr), large neutral amino acids (LNAA), Trp:LNAA ratio, free-Trp:Tyr ratio, total Trp:Tyr ratio, and plasma prolactin] (*P *> 0.05) with the exception of plasma free-Trp:LNAA ratio which was higher at 90 min and at exhaustion during the FC trial (*P *< 0.05).

**Conclusions:**

Neither brain 5-HT nor DA systems would appear to be implicated in the fatigue process when exercise is performed without significant thermoregulatory stress, thus indicating fatigue development during exercise in relatively cold temperatures to occur predominantly due to glycogen depletion.

## Background

Following the exclusion of caffeine from the World Anti-Doping Agency list of prohibited substances, there was an increased interest in freely using caffeine, particularly by endurance athletes, as an ergogenic aid supplement [1]. It was previously reported that caffeine, at doses of 3-9 mg.kg^-1 ^body mass, enhances performance by altering substrate availability; more specifically by promoting adipose tissue lipolysis and fatty acids oxidation from skeletal muscle which contributes in enhancing carbohydrate (CHO) sparing [2,3]. Recently however, a considerable amount of evidence has cast doubts over the CHO-sparing effect of caffeine during endurance exercise [e.g. [4,5]. In addition, caffeine has been shown to improve short duration high-intensity exercise performance where glycogen depletion is clearly not the primary cause of fatigue [e.g. [6,7]. Therefore, it is possible that the ergogenic effect of caffeine reflects a stimulant action on the CNS [8,9] rather than the traditional CHO-sparing effect during endurance exercise.

Animal studies, for example, suggest that caffeine has the potential to reduce brain serotonin (5-HT) synthesis by inhibiting tryptophan hydroxylase, the rate limiting enzyme of central 5-HT biosynthesis [10], and/or to reduce brain 5-HT:dopamine (DA) ratio by blocking adenosine α_1 _and α_2 _receptors within the CNS, which otherwise inhibit brain DA synthesis [8,11]. Consequently, one plausible explanation for the reduced effort perception observed following caffeine ingestion [12] may be due to the increased brain DA levels [8] and/or to the reduced brain 5-HT response [10]. This is consistent with the hypothesis that a high brain 5-HT:DA ratio may favour increased subjective effort and central fatigue, while a low 5-HT:DA ratio may favour increased arousal and central motivation [13,14].

Newsholme et al. [15] proposed that an increase in activity of 5-HT neurons in various brain regions such as the midbrain and hypothalamus may contribute to fatigue development during prolonged exercise, a mechanism commonly referred as the "central fatigue hypothesis". 5-HT is synthesised from the essential amino acid precursor tryptophan (Trp) and during periods of high 5-HT activity, the rate of 5-HT synthesis can be influenced by the uptake of Trp from plasma [16]. A rise in plasma free fatty acids (FFA) concentration displaces Trp from albumin raising the Trp fraction in plasma, thus increasing brain Trp uptake and arguably 5-HT synthesis [17,18]. Subsequently, the net effect of ingesting caffeine prior to exercise would be to increase central DA release and/or to counterbalance the high 5-HT:DA ratio reducing therefore effort perception induced by the exercise stress [14]. Consequently, the aim of the present study was to examine the relationship between peripheral modulators of brain 5-HT and DA function, perceptual responses and endurance performance during prolonged submaximal exercise to volitional fatigue, following caffeine co-ingested with a high fat meal in well-trained cyclists. The pre-exercise high fat meal was employed to imitate physiologically the metabolic effects of caffeine in an attempt to distinguish between the potential peripheral and/or central effects of caffeine.

## Methods

### Participants

Ten endurance-trained male cyclists [age 25 ± 6 years; height 1.82 ± 0.07 cm; body mass 74.34 ± 8.61 kg; maximal oxygen uptake (VO_2_max) 62 ± 5 ml‧kg^-1^‧min^-1^] volunteered to participate in the present study. All participants gave their written informed consent to take part in the study, which was approved by the local research ethics committee.

### Experimental design

The participants initially underwent ramp incremental exercise (15-20 W‧min^-1^) to the limit of tolerance using an electrically braked cycle ergometer (Bosch Erg-551 Forckenbecksti, Berlin, Germany) to determine VO_2_max and the maximal work rate. The participants were required to undertake three cycled exercise tests to exhaustion, at an ambient temperature of 10°C with 70% relative humidity, at ~73% of VO_2_max (a work-rate equivalent to 63% ± 5 of each individual's maximal work rate).

The participants underwent at least two familiarisation trials prior to the three exercise tests in order to become familiarised with the exercise protocol and experimental procedures. During the familiarisation period (i.e., 3 days prior to the second familiarisation trial) each participant's normal energy intake and diet composition were determined from weighted dietary intake data using a computerised version of the food composition tables of McCance and Widdowson (revised by Holland *et al*., [19]). Based on this information, subjects were prescribed a high (70%) CHO diet throughout the study period (for twelve consecutive days), intended to increase and maintain liver and muscle glycogen concentration before each of the main exercise trials [20]. The 70% CHO diet was isoenergetic with each participant's normal daily energy intake, and food items prescribed were based predominantly on each participant's normal diet.

Four hours prior to the first exercise test the participants consumed a standardised high CHO meal (Control trial: 90% of energy intake in the form of CHO). The control trial was always performed first and therefore, this trial was not included in the randomization, and hence in the statistical analysis. Four hours before the second and third exercise tests, the participants consumed a standardised high fat meal (1g fat‧kg^-1 ^body mass; 90% of energy intake in the form of fat). All experimental meals were isoenergetic and prepared by the same investigator. One hour before exercise following the high fat meals (second and third tests), participants ingested, in a cross-over double blind manner, capsules containing either caffeine (7.5 mg‧kg^-1 ^body mass; FC trials) or an equivalent amount of placebo (calcium carbonate; F trial). The participants, who were habitually moderate caffeine users (from none to two cups of coffee per day), were required to maintain normal training habits throughout the study period, but refrain from strenuous training and consumption of alcohol or caffeine-containing products 48 hrs prior to each exercise test.

### Procedures

All exercise tests were carried out between 16:00-21:00 h following a 4 h fast, where water was allowed *ad libitum*. Participants reported to the laboratory 1 1/2 h before the start of exercise, and on the two fat trials consumed capsules containing caffeine or placebo, 3 h after consuming the fat meal. Once body mass was measured, participants were seated comfortably with their right hand and forearm immersed for 15 min in water at 42-44°C, to achieve arterialization of the venous blood [21]. Following this, an 18 G venous cannula was introduced into a superficial vein on the dorsal surface of the heated hand and a resting blood sample was obtained. Further blood samples were obtained at 15 min intervals throughout exercise until the 90 min time-point and at exhaustion. Participants were transferred to the climatic chamber (ambient temperature 10.2 ± 0.2°C; relative humidity 69.8 ± 1.0%; air velocity of approximately 3.6 m.s^-1^) and began exercise within 1 min of entering. The exercise intensity and ambient temperature were chosen to induce fatigue that would be most likely due to muscle glycogen depletion rather than the result of some failure in the thermoregulatory system [22]. The cannula was kept patent by a slow (~0.5 ml.min^-1^) infusion of isotonic saline between samples during both experiments. Arterialization of the venous blood was maintained throughout exercise by heating the hand using an infrared lamp. The participants ingested 7.14 g‧kg^-1 ^and 2.14 g‧kg^-1 ^of water at rest and every 15 min throughout exercise, respectively. The participants were asked to maintain a pedal cadence of 60-80 rev‧min^-1 ^throughout the test; exhaustion was defined as the point at which the subject could no longer maintain the pedal cadence above 60 rev‧min^-1^

Expired gas was collected in Douglas bags for 5 min at rest, and thereafter 1 min collections were obtained every 15 min during exercise. Expired gases were analysed within 5 min of collection for oxygen uptake (VO_2_) (Servomex 570A, East Sussex, UK) and carbon dioxide production (VO_2_) (Servomex 1400 B4, East Sussex, UK), volume (dry gas meter, Harvard Apparatus Ltd., Hertfordshire, UK) and temperature (C6600 10-Channel Microprocessor, Comark, Hertfordshire, UK). All gas volumes were corrected to STPD. Barometric pressure was measured using a standard mercury barometer.

The participants were asked to rate ''shortness of breath'' (breathlessness/dyspnoea) and ''leg effort'' (leg exertion) using Borg's 6 - 20 RPE scale [23] every 10 min during exercise until exhaustion. Heart rate (Polar Sport Tester, Polar Electro Oy, Finland) was also recorded every 10 min during exercise until exhaustion. Following exercise, participants were weighed and loss of body mass was calculated, after correcting for water consumed during exercise. Time to exhaustion was recorded, but withheld from the participant until all trials had been completed and the participant had answered the post-intervention questionnaire. Participants were asked: (1) to predict the order of treatments received during the study; (2) to nominate the treatment they perceived produced their best performance; and (3) to indicate which trial they found the most difficult.

### Blood treatment and analysis

Blood (10 ml) was drawn into dry syringes and dispensed into tubes containing K_3_EDTA and the remaining into tubes containing no anticoagulant for later use. Duplicate aliquots (400 *μ*l) of whole blood from the K_3_EDTA tubes were rapidly deproteinized in 800 *μ*l of ice-cold 0.3 mol.l^-1 ^perchloric acid. After centrifugation, the supernatant was used for the measurement of glucose, lactate and pyruvate using standard enzymatic methods with spectrophotometric detection (Mira Plus, ABX Diagnostics, Montpellier, France). A further aliquot of blood was centrifuged and the plasma obtained was separated and used for the measurement of free fatty acids (colorimetric method, Roche Diagnostics GmbH, Germany) and concentrations of amino acids by HPLC using fluorescence detection and pre-column derivitisation with 18 *o*-phthalaldehyde (Hypersel Amino acid method, ThermoHypersil-Keystone, Runcorn, UK). Free-Trp was separated from protein-bound Trp by filtering plasma through 10,000 NMWL 'nominal molecular weight limit' cellulose filters (Ultrfree-MC filters, Millipore Corporation, USA) during centrifugation at 5000 g for 60 min at 4°C. Prior to centrifugation, filters were filled with a 95% O_2 _- 5% CO_2 _mixture in order to stabilize pH. The blood in tubes without anticoagulant was allowed to clot and then centrifuged; the serum collected was used for the measurement of prolactin (Prl) by sandwich magnetic separation assay (Technicon Immuno 1 System, Bayer Diagnostics, Newbury, UK).

### Statistical analysis

Data are expressed as the mean ± SD following a test for the normality of distribution. For data that violated the assumptions for parametric analyses (i.e. equality of variance and normality of distribution) non-parametric analyses was carried out and these data were expressed as the median (range). As all participants completed the control trial first and were subsequently assigned to the two fat trials in randomized order, statistical analysis was carried out on the two fat trials. Statistical analysis of the data was carried out using a two-factor analysis of variance (treatment × time) for repeated measures followed by Student's t-test for paired data, where necessary. Time to exhaustion was not normally distributed and was therefore analysed using the Wilcoxon signed rank test. Statistical significance was declared at *P *< 0.05.

## Results

### Time to Fatigue and ratings of perceived exertion

**Figure 1:**
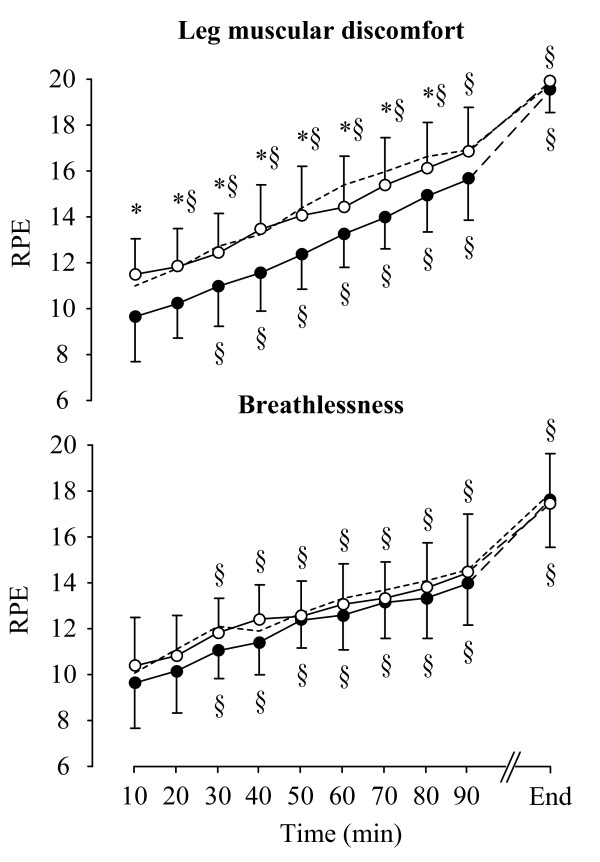
**Ratings of perceived exertion, for leg muscular discomfort (top panel) and breathlessness (bottom panel)**. *: indicates a significant difference between the F (white dots) and the FC (black dots) trials. ^§^: indicates significant differences within the trials compared with the 15 min time-point. The dash line indicates the Control trial. Values are presented as the mean ± SD.

Time to fatigue during constant-load exercise was similar between the two fat trials [(Control trial: 116(88-145) min; F trial: 122(96-144) min; FC trial: 127(107-176) min)]. Ratings of perceived leg exertion were significantly lower (F_(1,9) _= 11.985, *P *= 0.007) during constant-load exercise on the FC compared with the F trial while ratings of perceived breathlessness were not different between the trials (Figure 1). Six out of ten subjects ranked the FC as the easiest trial (one subject was unsure).

### Cardiopulmonary variables and fuel oxidation

O_2 _increased over time on both trials and it was higher on the FC trial compared with the F trial (F_(1,9) _= 7.980, *P *= 0.02) (Table 1). Minute ventilation (E) was significantly higher on the FC trial compared with F trial (F_(1,9) _= 10.917, *P *= 0.009) and there was a progressive increase in E and co_2 _over time on both fat trials; no differences in respiratory exchange ratio (RER) were found between F and FC trials (Table 1). Heart rate and total CHO and fat oxidation (FC trial: 371 ± 82g CHO, 77 ± 50g fat; F trial: 388 ± 90g CHO, 52 ± 23g fat; Control trial: 367 ± 87g CHO, 39 ± 23g fat) were not different between the F and FC trials.

**Table 1:** Cardiopulmonary variables.

		Exercise Time (min)						
		
Variables	Trials	Rest	15	30	45	60	75	90
O_2 _(L·min^-1^)	Control	.3 ± .04	3.2 ± 0.4	3.2 ± 0.4	3.4 ± 0.5	3.4 ± 0.5	3.5 ± 0.6	3.4 ± 0.4
	F	.3 ± .03	3.1 ± 0.4	3.2 ± 0.4^§^	3.2 ± 0.4	3.4 ± 0.4^§^	3.4 ± 0.5^§^	3.5 ± 0.5^§^
	FC	.4 ± .07	3.3 ± 0.3	3.4 ± 0.4	3.4 ± 0.5^§^	3.5 ± 0.5^§^	3.6 ± 0.5*^§^	3.6 ± 0.5^§^
								
CO_2 _(L·min^-1^)	Control	.3 ± .04	3.0 ± 0.5	3.0 ± 0.5	3.1 ± 0.5	3.1 ± 0.5	3.2 ± 0.7	3.1 ± 0.5
	F	.3 ± .03	3.0 ± 0.4	3.1 ± 0.4	3.1 ± 0.4	3.2 ± 0.4^§^	3.2 ± 0.4^§^	3.3 ± 0.5^§^
	FC	.3 ± .05	3.0 ± 0.3	3.1 ± 0.4	3.1 ± 0.4	3.2 ± 0.4	3.3 ± 0.5^§^	3.2 ± 0.4
								
E (L·min^-1^)	Control	8.0 ± 2	66 ± 1	69 ± 1	73 ± 1	74 ± 1	78 ± 1	76 ± 9.0
	F	8.0 ± 1	66 ± 1	68 ± 1	70 ± 1^§^	73 ± 1^§^	76 ± 1^§^	78 ± 14^§^
	FC	10 ± 2	70 ± 6	73 ± 8*^§^	75 ± 1*^§^	79 ± 1*^§^	81 ± 1*^§^	81 ± 10^§^
								
RER	Control	.89 ± .08	.95 ± .3	.95 ± .03	.94 ± .05	.94 ± .03	.93 ± .04	.93 ± .02
	F	.87 ± .10	.95 ± .3	.94 ± .03	.93 ± .04	.93 ± .03^§^	.93 ± .02	.91 ± .03^§^
	FC	.87 ± .07	.93 ± .4	.91 ± .03^§^	.91 ± .05	.91 ± .05	.90 ± .06	.88 ± .05^§^

Values are presented as the mean ± SD

*: Indicates a significant difference from the F trial at the same time-point.

^§^: Significant difference within the trials compared with the 15 min time-point.

*Note*: RER = Respiratory exchange ratio.

### Plasma amino acids, prolactin and blood metabolites

**Figure 2:**
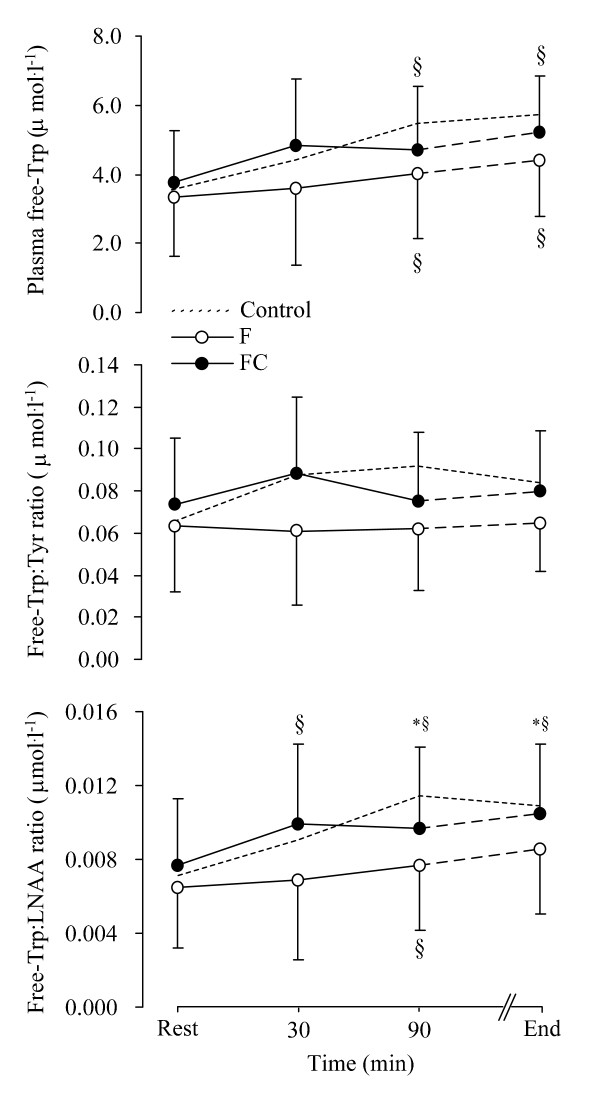
**Plasma free-Trp:LNAA ratio (bottom panel), free-Trp:Tyr ratio (middle panel) and plasma free-Trp (top panel)**. *: indicates a significant difference between the F (white dots) and the FC (black dots) trials. ^§^: indicates significant differences within the trials compared with the 15 min time-point. The dash line indicates the Control trial. Values are presented as the mean ± SD.

**Figure 3:**
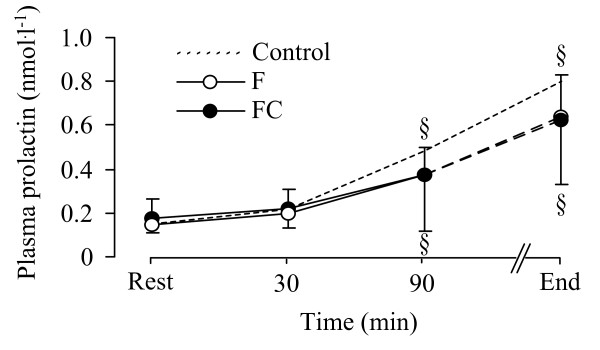
**Plasma prolactin responses between the F (white dots) and the FC (black dots) trials**. ^§^: indicates significant differences within the trials compared with the 15 min time-point. The dash line indicates the Control trial. Values are presented as the mean ± SD.

**Figure 4:**
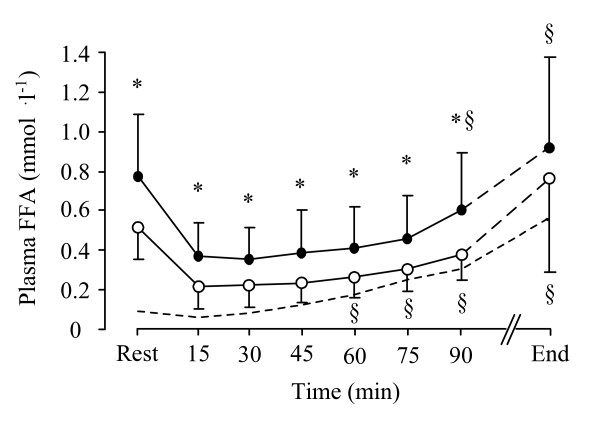
**Plasma FFA responses**. *: indicates a significant difference between the F (white dots) and the FC (black dots) trials. ^§^: indicates significant differences within the trials compared with the 15 min time-point. The dash line indicates the Control trial. Values are presented as the mean ± SD.

There were no significant differences between F and FC trials in total [Trp], [Tyr], [LNAA], total [Trp]:[LNAA] ratio and total [Trp]:[Tyr] ratio (Table 2). However, there was a tendency for plasma free-[Trp] (*P *= 0.064) and free-[Trp]:[Tyr] ratio (*P *= 0.066) to be higher on the FC relative to F trial (Figure 2). Plasma free-[Trp]:[Tyr] ratio did not change over time. Plasma free-[Trp] increased over time in both trials. The plasma free-[Trp]:[LNAA] ratio was significantly higher at 90 min and at exhaustion on the FC relative to F trial (*P *= 0.029) (Figure 2). The plasma [Prl] was not different between trials (Figure 3). The peak plasma [Prl] value was detected at exhaustion. A higher plasma [FFA] was found on the FC compared to the F trial (F_(1,9) _= 10.959, *P *< 0.01 *P *= 0.009) at rest and during exercise (Figure 4). Higher blood [glucose] (F_(1,9) _= 23.329, *P *< 0.001), [lactate] (F_(1,9) _= 13.823, *P *< 0.01) and [pyruvate] (F_(1,9) _= 35.262, *P *< 0.001) was found throughout exercise on the FC compared with the F trial (Table 3). There was no correlation between time to exhaustion and any of the other depended variables.

**Table 2:** Plasma amino acids concentrations.

		Blood collection time (min)			
Variables	Trials	Rest	30 min	90 min	End
Total [Trp] (μmol·l^-1^)	Control	38 ± 8	36 ± 7	39 ± 3	46 ± 9
	F	38 ± 7	39 ± 7^§^	43 ± 6^§^	42 ± 9
	FC	38 ± 7	39 ± 7	43 ± 9^§^	43 ± 7^§^
					
[Tyrosine] (μmol·l^-1^)	Control	54 ± 8	53 ± 7	61 ± 7	71 ± 8
	F	52 ± 3	58 ± 6^§^	65 ± 7^§^	68 ± 5^§^
	FC	51 ± 4	55 ± 6^§^	64 ± 8^§^	66 ± 7^§^
					
[LNAA] (μmol·l^-1^)	Control	500 ± 50	487 ± 35	486 ± 51	531 ± 60
	F	522 ± 46	532 ± 50	518 ± 45	518 ± 54
	FC	505 ± 40	499 ± 48	504 ± 48	506 ± 44
					
Total [Trp]:[LNAA] ratio	Control	.076 ± .013	.077 ± .012	.081 ± .009	.088 ± .016
	F	.072 ± .012	.074 ± .013	.083 ± .015^§^	.083 ± .021
	FC	.075 ± .012	.080 ± .013	.085 ± .013^§^	.085 ± .015^§^
					
Total [Trp]:[Tyrosine] ratio	Control	0.72 ± .15	0.69 ± .13	.064 ± .08	0.66 ± .11
	F	0.72 ± .14	0.68 ± .13^§^	0.67 ± .11	0.63 ± .15^§^
	FC	0.74 ± .17	0.72 ± .14	0.67 ± .14	0.65 ± .10^§^

Values are presented as the mean ± SD

^§^: Significant difference within the trials compared with the resting values.

**Table 3:** Blood glucose, lactate and pyruvate concentrations for each of the three trials.

		Blood collection time (min)							
Variables	Trials	Rest	15	30	45	60	75	90	End
[Glucose] (mmol·L^-1^)	Control	4.9 ± 0.9	3.8 ± 0.4	4.1 ± 0.3	4.2 ± 0.4	4.0 ± 0.4	3.9 ± 0.4	3.9 ± 0.5	4.1 ± 1.0
	F	4.7 ± 0.6	4.1 ± 0.5	4.4 ± 0.4^§^	4.3 ± 0.3	4.1 ± 0.3	3.9 ± 0.3	3.8 ± 0.4	3.8 ± 0.4
	FC	4.7 ± 0.3	4.6 ± 0.4	4.8 ± 0.3*	4.8 ± 0.4*	4.7 ± 0.4*	4.4 ± 0.4*	4.3 ± 0.3*^§^	4.1 ± 0.5*^§^
									
[Lactate] (mmol·L^-1^)	Control	0.8 ± 0.2	3.6 ± 1.9	3.4 ± 2.1	3.5 ± 2.2	3.6 ± 2.1	3.8 ± 2.4	3.5 ± 1.8	4.5 ± 1.8
	F	0.8 ± 0.3	3.4 ± 0.9	3.1 ± 1.1	3.0 ± 1.3^§^	2.9 ± 1.3^§^	2.9 ± 1.2^§^	3.1 ± 1.2	4.1 ± 2.0
	FC	0.8 ± 0.2	4.1 ± 1.5*	4.0 ± 1.8*	3.9 ± 1.9*	3.8 ± 1.9*	3.9 ± 1.9*	3.9 ± 1.8*	5.1 ± 2.1*
									
[Pyruvate] (*μ*mol·L^-^)	Control	157 ± 33	230 ± 46	218 ± 50	221 ± 49	224 ± 51	228 ± 48	234 ± 53	254 ± 61
	F	159 ± 33	235 ± 49	223 ± 58^§^	218 ± 53	212 ± 57	215 ± 44	216 ± 47	219 ± 46
	FC	163 ± 41	256 ± 52	252 ± 58*	250 ± 57*	245 ± 57*	237 ± 63	239 ± 61	234 ± 51

Values are presented as the mean ± SD

*: Indicates a significant difference from the F trial at the same time-point.

^§^: Indicates a significant difference within the trials compared with the 15 min time-point.

### Reported side effects

Four out of the ten subjects experienced slight gastrointestinal discomfort; three following the high fat meal with caffeine and one following the high fat meal alone. One subject experienced more severe side effects following the high fat meal and caffeine ingestion 30 min following exercise. These effects included loss of consciousness, dizziness, abdominal pain, nausea and vomiting. These effects disappeared shortly after the experience.

## Discussion

The present study examined the relationship between the putative modulators and indices of brain serotonergic and dopaminergic function, effort perception and endurance exercise performance in a relatively cold (10°C) environment following caffeine co-ingestion with a high fat meal in well-trained humans. The results presented here do not support any significant involvement of the putative modulators of brain serotonergic and dopaminergic function in the exercise fatigue process during submaximal constant-load exercise at low ambient temperatures. This lack of involvement of the putative modulators of 'central fatigue' was observed despite a significant reduction in effort perception following caffeine ingestion. It is difficult however, to explain why the subjects in the present experiment perceived it easier to exercise with caffeine than without, particularly when one considers the accompanying elevation in blood [lactate], O_2_, and E that typically would be expected to augment, rather than attenuate effort perception [23]. It is possible that caffeine may attenuate effort perception by lowering the pain threshold through direct central neural effects [9], but the exact mechanism remains unclear.

Caffeine at the micromolar levels utilised in the present study has been shown to cross the blood brain barrier (BBB) with the potential to serve as a competitive antagonist of adenosine [11]. The net effect would be to increase central DA release by antagonising the inhibition of adenosine α_1 _and α_2 _receptors on DA activity, thus reducing effort perception induced by the exercise-stress [8]. This was consistent with the hypothesis that a high 5-HT:DA ratio may favour increased effort perception and central fatigue, while a low 5-HT:DA ratio may favour increased arousal and motivation [13,14]. Studies using rats for example, found a reduction in brain 5-HT synthesis and in the 5-HT:DA ratio, and an improvement in exercise performance after direct intracerebroventicular caffeine injection [8]. Similar results were found after an attenuation of the enzyme Trp hydroxylase through caffeine administration [10]. In the present experiment however, although effort perception was significantly lower with caffeine exercise performance was not different between the trials. This result suggests a mismatch between effort perception responses and endurance performance during exercise in 10°C following caffeine co-ingested with a high fat meal. In addition, a disparity was observed between effort perception and peripheral precursors of brain 5-HT synthesis. Although plasma free-[Trp]:[LNAA] ratio was higher with caffeine throughout exercise (*P *= 0.029) (Figure 2), effort perception was significantly lower in the same trial.

The failure of caffeine to significantly affect brain serotonergic function during exercise in the present study is further reflected by the lack of difference in plasma [Prl] (the brain 5-HT and DA metabolic-interaction marker) between the trials. Previous studies have shown that Ketanserin, a 5-HT antagonist drug, reduced Prl release during graded exercise to exhaustion [24,25]. A further study reported that Trp infusion reduced exercise performance and caused an earlier elevation in plasma [Prl] relative to placebo or glucose infusion [26]. In addition, evidence suggests that Prl release is mainly under the control of the central serotonergic system and/or under the hypothalamic 5-HT and DA metabolic interaction [27]. DA for example, has been suggested to be the major Prl-secretion inhibitor factor [28], and 5-HT injection or its agonist precursors and re-uptake inhibitors have been found to increase hypothalamic Prl release and, hence, plasma [Prl] [29]. Consequently, we hypothesised that if caffeine could directly attenuate brain 5-HT synthesis [10] and/or enhance DA release [8], Prl secretion would be expected to be lower during the exercise trial involving caffeine. The finding of lack of difference in plasma [Prl] between trials may imply that caffeine contributes in reducing effort perception (via a direct brain dopaminergic-mediated effect) but it may not be effective enough to alter neuroendocrine Prl secretion, particularly in trained humans and when exercise is carried out in relatively cold environment. Alternatively, circulating Prl levels may not be a sensitive marker of brain 5-HT [24,25].

Previous studies have demonstrated that elevation in plasma [FFA] displaces Trp from binding to albumin and consequently increases the free-Trp:LNAA ratio into the plasma [17,18,30,31]. Since Trp and the other LNAAs share the L-system carrier for crossing the BBB, the elevation in plasma free-Trp:LNAA ratio may favour brain Trp uptake and potentially increase brain 5-HT synthesis [32], and hence central fatigue [15,33]. A recent study using analbuminaemic rats has shown an improvement in exercise performance after reducing brain Trp uptake by blocking the L-system carrier using 2-aminobicyclo[2,2,1]heptane-2-carboxylic acid, a specific inhibitor of the L-system transporter [34]. Conversely, intracerebroventricular Trp injection in the same species was found to increase  and reduce mechanical efficiency and exercise performance in rats [35]. In the present experiment, the free-[Trp]:[LNAA] ratio was significantly higher following caffeine ingestion. This effect may have attributed to the action of caffeine in elevating adipose tissue lipolysis and thus plasma [FFA], results that are consistent with several previous reports [e.g. [2,3]]. This effect, in conjunction with a reduced effort perception following caffeine ingestion could reflect the two opposing actions of the high fat meal and caffeine interventions. The former potentially increasing 5-HT function and subsequently effort perception [36], and the latter increasing DA function, hence reducing effort perception [8,14]. However, although caffeine may have effectively reduced effort perception by possibly elevating brain DA function exercise performance was not enhanced.

Total CHO and fat oxidation were not different between F and FC trials. These results help confirm the lack of significant involvement of the brain serotonergic and dopaminergic modulators during this type of exercise. These results also support the role of glycogen depletion in fatigue development during prolonged exercise in well-trained humans in relatively cold environments [22]. However, the role of elevated brain DA levels in the reduction of perceptual responses and improvement in performance during fatiguing exercise in a warm environment is further supported by recent studies. Watson et al. [37] for example, examined the effects of DA and norepinephrine (NE) reuptake inhibitors in a temperate or in a warm condition. These authors suggested that DA reuptake inhibitors was able to reduce effort perception and enhance performance in the heat by superseding hyperthermia-induced inhibitory signals within the central nervous system responsible to terminate exercise trial. Similarly, Roelands et al. [38] examined the effects of methylphenidate, a DA reuptake inhibitor, on exercise performance suggesting that this drug improve 30 minutes time-trial in the heat, but not in normal environmental temperature. As it was mentioned above, in the present study caffeine did not appear to influence substrate utilisation, consequently, no improvement in exercise performance could be reasonably expected, as it is well established that fatigue during prolonged exercise at 10°C is due to glycogen depletion [22]. The improvements therefore, in endurance exercise performance observed in previous caffeine studies are unlikely to be associated with glycogen depletion, unless caffeine ingestion altered substrate utilisation. This is the reason why in the present study a time to fatigue protocol, which glycogen depletion could be achieved, was employed. Due to the experiment design, in the present study we were able to examine both the metabolic (peripheral) and central (brain neurotransmission modulators and indices) effects of caffeine during prolonged exercise.

Based on the results presented here, one could argue that the lack of performance improvement following caffeine ingestion in conjunction with the reduced effort perception observed is due to either the time to peak plasma caffeine concentration or to individual differences in caffeine uptake. We do not think however, that time to peak plasma concentration had any significant effect on the results since all subjects followed exactly the same experimental procedure prior to each exercise trial. On the other hand, the intra-individual differences in caffeine uptake may elevate type II statistical error in the present and perhaps in other previous studies where caffeine was used as a treatment. This could be evident, if we take into consideration that there may be "responders" and "non-responders" to various drugs including perhaps caffeine. In a psychophysiological study for example, where the differences between the "responders" and "non-responders" to brain neurotransmission manipulating drug (e.g. brofaromine and fluvoxamine) therapy were examined, it was suggested that some physiological responses (e.g. heart rate and blood pressure responsiveness) to the drugs were different between the two groups, being higher in the "non-responders" than the "responders" to the drug group [39]. Similarly, Kampf-Sherf et al. [40] examined the physiological responses to selective serotonin reuptake inhibitors (SSRI) treatment to depressed patients and they suggested that only two third of patients with major depression have shown physiological responses to antidepressants such as SSRI. In a previous also study, the drug amynophylline was used as a "vehicle" to test the physiological responses as well as adenosine receptors to the drug [41]. These authors suggested that there are "responders" and "non-responders" to the amynophylline and this might be explained by genetic differences in some elements of the adenosine signalling pathway in humans [41]. Consequently, to minimise the effect of this confounding variable on future exercise performance studies, studies may be necessary to try and identify "responders" and "non-responders" to caffeine prior to starting the experimental trials.

## Conclusions

In conclusion, brain serotonergic and dopaminergic systems are unlikely to be implicated in the fatigue process when exercise is performed without significant thermoregulatory stress, thus enabling fatigue development during endurance exercise to occur predominantly due to glycogen depletion. Consequently, it could be suggested that when artificial elevation in plasma FFA occurs, caffeine does not improve endurance performance either through its potential peripheral metabolic pathway or via its possible central mediated effects (i.e. enhancement of brain dopaminergic system). For practical application purposes we would like to suggest that under the environmental circumstances that our experiment was executed, although caffeine was not found to significantly improve endurance performance, we could recommend that a pre-exercise caffeine ingestion may contribute to enable athletes a) to train with more motivation for progressively achieving elevation or maintenance in their performance and b) to compete with more enthusiasm to the limits of tolerance.

## Competing interests

The authors declare that they have no competing interests.

## Authors' contributions

MH was the primary author of the manuscript and participated in the design of the study and carried out the data collection, data analysis, statistical analysis and interpretation of the results. LK played an important role in study design, data collection and data interpretation and manuscript preparation. YP played an important role in study design, data collection and interpretation and study coordination. All authors read and approved the final manuscript.
